# Targeting Acne Bacteria and Wound Healing In Vitro Using *Plectranthus aliciae*, Rosmarinic Acid, and Tetracycline Gold Nanoparticles

**DOI:** 10.3390/ph15080933

**Published:** 2022-07-28

**Authors:** Isa A. Lambrechts, Velaphi C. Thipe, Kattesh V. Katti, Vusani Mandiwana, Michel Lonji Kalombo, Suprakas Sinha Ray, Rirhandzu Rikhotso, Arno Janse van Vuuren, Tenille Esmear, Namrita Lall

**Affiliations:** 1 Department of Plant and Soil Sciences, Faculty of Natural and Agricultural Sciences, University of Pretoria, Hatfield, Pretoria 0002, South Africa; u29021759@tuks.ac.za (I.A.L.); tenille.esmear@gmail.com (T.E.); 2 Department of Radiology, Institute of Green Nanotechnology, University of Missouri Columbia, Columbia, MO 65212, USA; vctqnf@health.missouri.edu (V.C.T.); kattik@health.missouri.edu (K.V.K.); 3 Chemical Cluster, Centre for Nanostructures and Advanced Materials, Council for Scientific and Industrial Research, Pretoria 0184, South Africa; vusy.mandi@gmail.com (V.M.); lkalombo@csir.co.za (M.L.K.); 4 DST/CSIR National Centre for Nanostructured Materials, Council for Scientific and Industrial Research, Pretoria 0184, South Africa; rsuprakas@csir.co.za (S.S.R.); rrikhotso@csir.co.za (R.R.); 5 Centre for High-Resolution Transmission Electron Microscopy, Nelson Mandela University, Port Elizabeth 6001, South Africa; arno.jansevanvuuren@mandela.ac.za; 6 School of Natural Resources, College of Agriculture, Food and Natural Resources, University of Missouri, Columbia, MO 65211, USA; 7 College of Pharmacy, JSS Academy of Higher Education and Research, Mysuru 570015, India

**Keywords:** gold nanoparticles, *Plectranthus aliciae*, rosmarinic acid, tetracycline, wound healing, antibiotic resistance, *Cutibacterium acnes*, *Staphylococcus epidermidis*

## Abstract

Gold nanoparticles from plant extracts and their bioactive compounds to treat various maladies have become an area of interest to many researchers. Acne vulgaris is an inflammatory disease of the pilosebaceous unit caused by the opportunistic bacteria *Cutibacterium acnes* and *Staphylococcus epidermis*. These bacteria are not only associated with inflammatory acne but also with prosthetic-implant-associated infections and wounds. Studies have hypothesised that these bacteria have a mutualistic relationship and act as a multispecies system. It is believed that these bacteria form a multispecies biofilm under various conditions and that these biofilms contribute to increased antibiotic resistance compared to single-species biofilms. This study aimed to investigate the antibacterial and wound healing potential of synthesised gold nanoparticles (AuNP_s_) from an endemic South African plant, *Plectranthus aliciae* (AuNP_PAE_), its major compound rosmarinic acid (AuNP_RA_) and a widely used antibiotic, tetracycline (AuNP_TET_). Synthesised gold nanoparticles were successfully formed and characterised using ultraviolet–visible spectroscopy (UV–vis), dynamic light scattering (DLS), Fourier transform infrared spectroscopy (FTIR), zeta potential (ζ-potential), high-resolution transmission electron microscopy (HRTEM), and selected area electron diffraction (SAED), and they were investigated for stability under various biological conditions. Stable nanoparticles were formed with ζ-potentials of −18.07 ± 0.95 mV (AuNP_PAE_), −21.5 ± 2.66 mV (AuNP_RA_), and −39.83 ± 1.6 mV (AuNP_TET_). The average diameter of the AuNP_s_ was 71.26 ± 0.44 nm, 29.88 ± 3.30 nm, and 132.6 ± 99.5 nm for AuNP_PAE_, AuNP_RA_, and AuNP_TET_, respectively. In vitro, biological studies confirmed that although no antibacterial activity or biofilm inhibition was observed for the nanoparticles tested on the multispecies *C. acnes* and *S. epidermis* systems, these samples had potential wound closure activity. Gold nanoparticles formed with rosmarinic acid significantly increased wound closure by 21.4% at 25% *v*/*v* (≈29.2 µg/mL) compared to the negative cell control and the rosmarinic acid compound at the highest concentration tested of 500 µg/mL. This study concluded that green synthesised gold nanoparticles of rosmarinic acid could potentially be used for treating wounds.

## 1. Introduction

Acne vulgaris is a disease of the pilosebaceous unit. Patients with acne vulgaris have confirmed that the disease affects their quality of life both socially and psychologically. Acne vulgaris develops when an abnormal shedding of the skin blocks the pilosebaceous unit. Overproduction of sebum in the pilosebaceous unit results in skin distention and acts as a carbon and nutrient source for acne-causing bacteria. Acne-causing bacteria proliferate in the sebum releasing various pathogenic enzymes that trigger an inflammatory response resulting in scarring and postinflammatory hyperpigmentation [1].

The skin microbiome consists of various bacterial species protecting the skin and the host from the external environment and other pathogenic bacteria. *Cutibacterium acnes* (anaerobic, aerotolerant) and *Staphylococcus epidermis* (aerobic) are Gram-positive bacteria that form part of the skin′s normal microbiota. These commensal bacteria are found abundantly in the skin and protect the host from pathogens. However, shifts in the external and skin environment result in dysbiosis of these opportunistic commensal bacteria. This shift allows these opportunistic bacteria to flourish, causing skin-related maladies such as acne vulgaris. *Cutibacterium acnes* and *S. epidermidis* are opportunistic bacteria that have been associated with maladies such as acne vulgaris, prosthetic-implant-associated infections, and wounds. Both *C. acnes* and *S. epidermidis* have been isolated from acne lesions either alone or in combination. It is hypothesised that these bacteria may exist in a mutually beneficial relationship and could have a combined bacterial effect that results in the formation of a multispecies biofilm under certain conditions. Biofilm formation of *C. acnes* and *S. epidermidis* contributes to antibiotic resistance observed in acne vulgaris, prosthetic-implant-associated infections, and wounds [2,3,4,5,6,7].

The antibiotic resistance of *C. acnes* and *S. epidermidis* results from the bacteria forming a protective biofilm. The biofilm protects these bacterial communities from their external environment, making them less susceptible to antibiotics treatments than planktonic bacteria, which are much more susceptible without this protective layer. The thick polymetric extracellular matrix of the biofilm structure prevents antibiotics from inhibiting the bacteria within [1,4,8]. Antibiotic resistance results in longer treatment times that could be life-threatening to the patient [3,9]. The World Health Organisation has recognised antibiotic resistance as a global threat to public health and the economy. Tetracycline is an antibiotic frequently used topically and orally to treat acne vulgaris and other infections such as wounds and implant-associated infections. The side effects of tetracycline include tooth discolouration and contact dermatitis, and it affect the gut microflora. Other acne treatments are available to treat acne vulgaris. However, these treatments have severe side effects, including contact dermatitis that can further harm the skin, increase photosensitivity, cause mental health issues, hearing loss, congenital disabilities, and vestibular dysfunction that can cause nausea. New treatments are required to overcome the mechanisms that result in antibiotic resistance and have fewer side effects [7,10,11,12,13]. 

*Plectranthus aliciae* (Codd) Van Jaarsv. & T.J.Edwards previously classified as *Plectranthus madagascariensis* (Pers.) Benth. var. aliciae Codd is an endemic South African plant. Traditionally, the Zulu and Xhosa communities use this species to treat respiratory ailments such as coughs and asthma, colds and flu, and skin-related maladies such as wounds and scabies. A study on the acetonic extract of *P. aliciae* confirmed that the species have antibacterial activity. *Plectranthus madagascariensis* displayed antibacterial activity against *S. epidermidis*, known to be associated with inflammatory acne and wounds. Several compounds have been isolated from *Plectranthus madagascariensis* and its varieties, such as *P. aliciae,* with antibacterial and potentially wound healing properties. One of these isolated compounds is rosmarinic acid. Rosmarinic acid has shown analgesic and anti-inflammatory activity and could potentially contribute to wound healing [14,15,16,17]. 

Nanotechnology has become an area of interest as a potential solution to overcome bacterial resistance. Due to the small size of nanoparticles compared to proteins and their large surface-to-volume ratio, nanoparticles can accommodate many functional groups, making them ideal candidates for targeting resistant bacteria. Studies have shown that nanoparticles capped with various compounds can pass through the bacterial envelope and inhibit bacteria due to their size. The biologically inert nature of gold and the increased biological activity of gold nanoparticles (AuNPs) makes this metal ideal for targeting antibiotic-resistant bacteria. AuNPs capped with various antibiotics and compounds for their antibacterial activity have undergone extensive research. Various factors play a role in the antibacterial activity of AuNPs. Research studies have revealed AuNPs with diameter sizes ranging between 2 and 52 nm to have noteworthy antibacterial activity. The surface charge of the AuNPs also plays a role in antibacterial activity, with positive surface charged AuNPs interacting with the negative electrostatic charges of the bacterial cell surface, inferring antibacterial activity. Furthermore, AuNPs capped with negatively charged functional groups have shown no antibacterial activity [8,18]. Nanoparticles could potentially provide a safe and effective alternative to current treatments. 

The present study aimed to determine whether the gold nanoparticles synthesised using *Plectranthus aliciae*, rosmarinic acid, and tetracycline show similar antimicrobial inhibition of biofilm development and wound healing activity compared to that of the extract and compounds alone. 

## 2. Results and Discussion

### 2.1. Ultraviolet–Visible Spectroscopy and Stability of AuNPs

UV–vis spectroscopy is widely used to characterise AuNPs based on their surface plasmon resonance (SPR). SPR is defined as the absorption band measured through UV–vis spectroscopy due to free conduction electrons associated with AuNPs, that oscillate when exposed to light. UV–vis spectroscopy is a nondestructive method to measure the aggregation of nanoparticles in a suspension and is widely used to determine the stability of nanoparticles. Therefore, UV–vis detects changes in transmitted light brought on by light scattering because of the turbidity of a suspension. Turbidimetry is a technique widely used by researchers to measure the aggregation of nanoparticles. UV–vis is used to determine the stability of the AuNPs in various biological solutions and, depending on the UV spectra, give information on the stability, diameter, and size distribution of the nanoparticles. The stability of AuNPs in biological systems is vital to ensure optimal activity under various physiological test conditions [19,20,21].

The stability of the AuNPs was evaluated in vitro in various buffers and media to mimic in vivo physiological and in vitro biological testing conditions. This information provides valuable insight into how the synthesised nanoparticles react under various conditions. Figure S1 (Supplementary Materials) shows the UV–vis absorption spectra of *Plectranthus aliciae* ethanolic extract gold nanoparticles (AuNP_PAE_), rosmarinic acid gold nanoparticles (AuNP_RA_)_,_ and tetracycline gold nanoparticles (AuNP_TET_) in various biological solutions. The absorbance intensity of the distilled water (Aq. dest.) UV–vis spectra for AuNP_PAE_ were around 539 nm and for AuNP_RA_ and AuNP_TET_ around 523 nm. Absorption peaks at low wavelengths indicate small spherical AuNP [19]. The maximum absorption of AuNP_RA_ could explain the small particle size observed using a dynamic light scattering (DLS) analysis (Section 2.5). Although AuNP_TET_ had a low absorption maximum at 523 nm, the particle sizes of these nanoparticles were variable, as indicated by the large standard deviation observed with DLS analysis (Section 2.5). 

AuNP_PAE_ was considered to be stable in the various biological solutions. Low stability was observed for AuNP_PAE_ in a 0.5% cysteine solution and brain heart infusion (BHI) broth. BHI broth has an overall pH of 7.4 ± 0.2 at room temperature; however, AuNP_PAE_ was stable at a pH of 7 in NaCl, an ingredient in BHI broth. The reduction in stability in BHI broth could be due to various factors that could neutralise the surface charge of the gold nanoparticles, causing aggregation. Studies have shown that glucose, an ingredient of BHI broth, could contribute to AuNP aggregation. AuNPs oxidise glucose to hydrogen peroxide and gluconic acid, causing a change in pH [22,23]. The change in pH could affect the stability of the AuNPs, as observed in the UV–vis spectral graphs for AuNP_RA_ and AuNP_TET_ (Figure S1B,C, Supplementary Materials). Another potential contributing factor to the low stability of the gold nanoparticles in BHI broth could be due to the presence of amino acids. Calf brain and pig heart are the main ingredients of BHI broth resulting in a media-rich in amino acids. A study performed on the aggregation of AuNPs in solutions containing certain amino acids showed that they contribute to the aggregation of AuNPs at a pH of 7. This could further explain the instability of the various AuNPs in the 0.5% cysteine solution [24].

AuNP_RA_ was stable in Aq. dest., 0.5% bovine serum albumin (BSA), and Dulbecco′s Modified Eagle Medium (DMEM), although an absorbance maxima shift was observed after 48 h. However, AuNP_RA_ was unstable in various pH solutions and displayed minimal stability at a pH of 10 for up to 24 h before a shift in the absorbance maxima was observed. AuNP_TET_ was stable for up to 72 h, whereafter the stability of the AuNPs slightly decreased. 

All AuNP samples were highly stable in DMEM, which is used to maintain cell cultures for cytotoxicity and wound healing in in vitro assays (Section 2.7 and Section 2.8). The stability of the AuNPs in DMEM could be due to the presence of 10% foetal bovine serum (FBS) in the media. A study on silver nanoparticles confirmed that DMEM, which had not been supplemented with 10% FBS, resulted in more unstable nanoparticles when compared to complete DMEM media. Studies have confirmed that certain proteins prevent aggregation of the AuNPs by forming a protein corona, contributing to abundant stable AuNPs. Furthermore, since FBS contains approximately 2.5 mg/mL of BSA, this could further explain the stability of the tested AuNPs in 0.5% BSA and DMEM [25,26,27,28].

### 2.2. Fourier Transform Infrared Spectroscopy (FTIR) Analysis 

**Figure 1:**
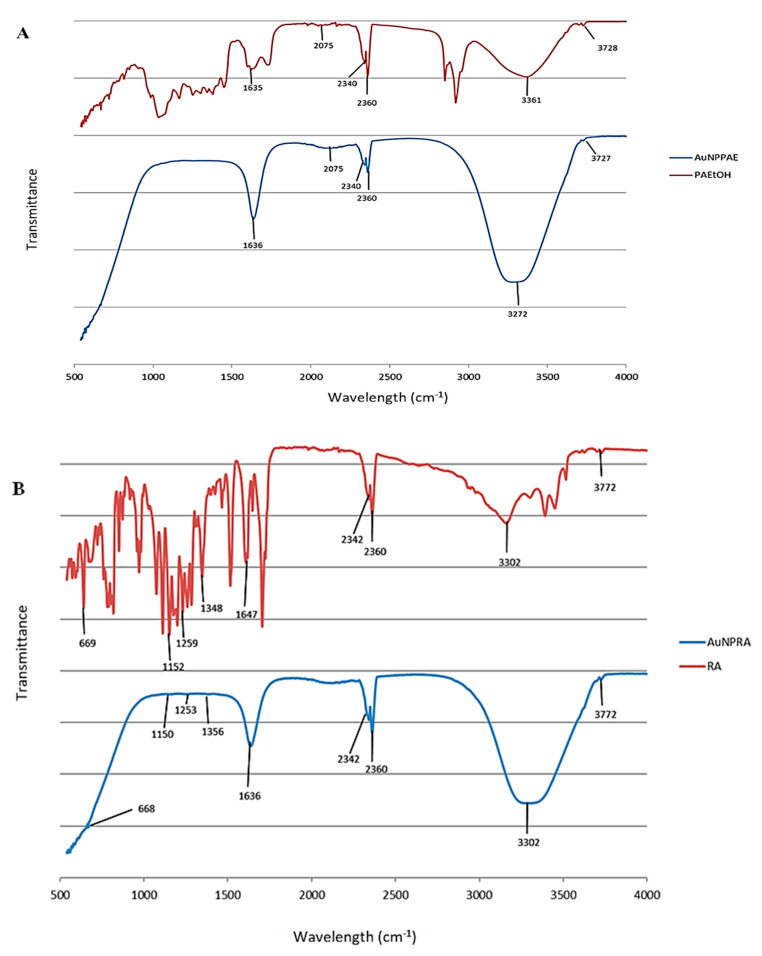

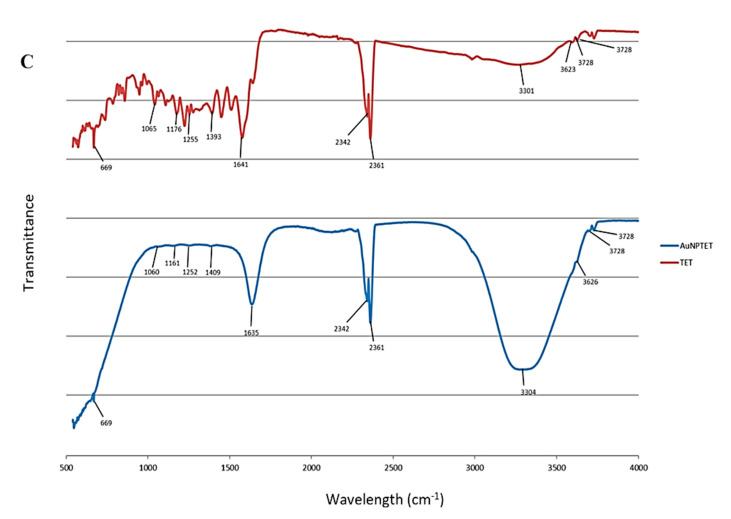
Fourier transform infrared spectra for (**A**) *Plectranthus aliciae* ethanolic extract (PAE) and *Plectranthus aliciae* ethanolic extract gold nanoparticles (AuNP_PAE_); (**B**) rosmarinic acid (RA) and rosmarinic acid gold nanoparticles (AuNP_RA_); (**C**) tetracycline (TET) and tetracycline gold nanoparticles (AuNP_TET_).

FTIR detects functional groups corresponding to the plant extract present in synthesised AuNPs. The functional groups capping the AuNPs are observed as peaks in the spectra [29]. The spectra of the synthesised AuNPs were compared to their respective controls that were not formulated into nanoparticles by reducing the gold salt. The peaks observed between 2360 and 2342 cm^−1^ in all figures are due to CO_2_ in the atmosphere (Figure 1A–C, Table S1). 

Figure 1A and Table S1 show the FTIR spectra of *Plectranthus aliciae* ethanolic extract (PAE) and AuNP_PAE_. Strong absorption bands were observed at 3361 cm^−1^ and 3272 cm^−1^ that could be attributed to N-H stretching, 2075 cm^−1^ assigned to N = C = S stretching, and 1635 cm^−1^ and 1636 cm^−1^ attributed to C = C or N-H bending for PAE and AuNP_PAE_, respectively. Rosmarinic acid was not a major capping agent during the formation of AuNP_PAE_ due to the nature of the functional groups of PAE that capped the AuNPs [30,31].

Major absorption bands for rosmarinic acid (RA) and AuNP_RA_ were observed at 3302 cm^−1^, which could be assigned to O-H stretching and 1647 cm^−1^ and 1636 cm^−1^ assigned to C=C stretching for RA and AuNP_RA,_ respectively. Minor peaks were observed at 1348 cm^−1^ and 1356 cm^−1^ assigned to O-H bending, 1259 cm^−1^ and 1253 cm^−1^ assigned to C-O stretching, 1152 cm^−1^ and 1150 cm^−1^ assigned to C-O stretching, and 669 cm^−1^ and 668 cm^−1^ assigned to C=C bending for RA and AuNP_RA,_ respectively (Figure 1B, Table S1). These infrared bands could be attributed to the functional groups observed in the chemical structure of rosmarinic acid [30,31].

Major peaks were observed for tetracycline (TET) and AuNP_TET_ with the FTIR spectra at 3301 cm^−1^ and 3304 cm^−1^ that could be assigned to N-H stretching 1641 cm^−1^ and 1635 cm^−1^ assigned to C = C or N-H bending. Minor peaks were observed at 1393 cm^−1^ and 1409 cm^−1^ assigned to O-H bending, 1252 cm^−1^ and 1255 cm^−1^ assigned to C-O stretching, 1161 cm^−1^ and 1176 cm^−1^ assigned to C-N stretching, 1060 cm^−1^ and 1065 cm^−1^ assigned to C-O stretching, and 669 cm^−1^ assigned to C = C bending for TET and AuNP_TET_, respectively (Figure 1C, Table S1). These infrared bands could be attributed to the functional groups observed in the chemical structure of tetracycline [30,31].

### 2.3. High-Resolution Transmission Electron Microscopy 

**Figure 2:**
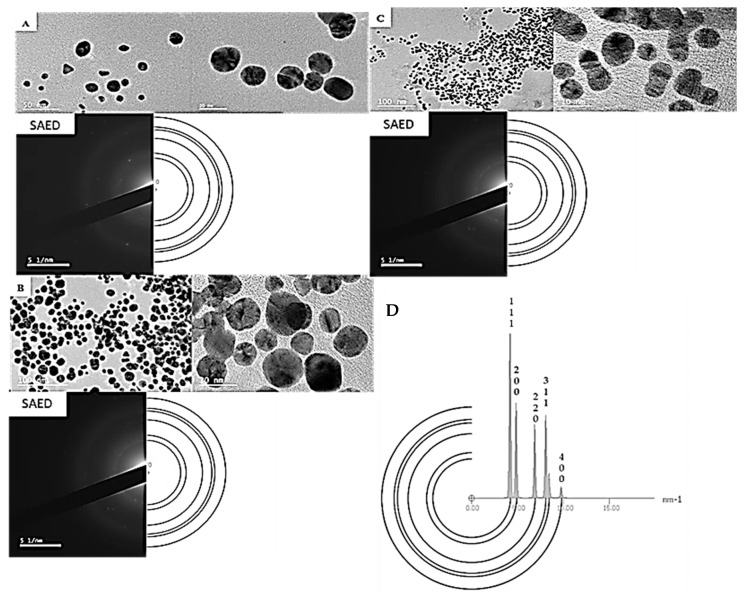
High-resolution transmission electron microscopy (HRTEM) images depicting clustering, lattice fringes, and selected area electron diffraction (SAED) patterns of biosynthesised gold nanoparticles (AuNP) compared to a simulated diffraction pattern of Au. (**A**) biosynthesised *Plectranthus aliciae* AuNPs; (**B**) biosynthesised rosmarinic acid AuNPs; (**C**) biosynthesised tetracycline AuNPs; (**D**) SAED pattern for the crystal of Au serving as a simulated diffraction pattern.

High-resolution transmission electron microscopy (HRTEM) was conducted to obtain the selected area electron diffraction (SAED) patterns, measure lattice fringes, and the overall shape of the AuNPs. Due to limited sample availability, the estimated d-spacing values were calculated from the SAED pattern for all the samples compared to the crystalline structure of Au, which represented all frequently occurring d-spacings in the crystal. The HRTEM images and SAED patterns of the gold nanoparticles AuNP_PAE_, AuNP_RA,_ and AuNP_TET_ are shown in Figure 2. Spherically shaped nanoparticles were mainly observed for AuNP_PAE_ with some triangular shapes (Figure 2A). These spherically shaped AuNP_PAE_ indicate a good capping and stabilisation of the AuNP by the plant extract components [32]. The HRTEM images of AuNP_RA_ (Figure 2B) and AuNP_TET_ (Figure 2C) confirmed that the nanoparticles were mainly spherically shaped, with some cluster formation observed. Clustering of these AuNP could be due to the functional groups capping the AuNPs. This clustering can be due to the acidic functional groups, such as carboxylic (>C=O) groups of rosmarinic acid and the basic amine functional groups of tetracycline [33]. Bright circular rings of the SAED pattern and clear lattice fringes from the HRTEM images confirmed the crystalline nature of AuNP_PAE,_ AuNP_RA,_ and AuNP_TET_. The d-spacings of the formed AuNP′s can be observed in Figure 2 compared to the simulated diffraction pattern of crystalline Au. 

### 2.4. Zeta (ζ) Potential 

Zeta potential is a measure of nanoparticle surface charge and indicates the nanoparticles’ long-term stability in a suspension. All the AuNPs in the present study had a negative zeta potential ranging from −18 to −40 mV. The higher the negative value of the AuNPs, the higher the stability of the NPs, and the lesser agglomeration and precipitation can be expected. A ζ-potential in the range of −16 to −30 mV is indicative of the beginning of dispersion, and a ζ-potential between −30 and −40 indicates medium stability [34]. The AuNPs, AuNP_PAE_, AuNP_RA_, and AuNP_TET_ had ζ-potential values of −18.07 ± 0.95 mV, −21.5 ± 2.66 mV, and −39.83 ± 1.6 mV, respectively. These results indicate that the AuNPs formed were stable. The colloidal stability of AuNP_RA_ was in agreement with previously formed AuNP_RA_ with a ζ-potential of −24.09 ± 3.97 mV. The researchers noted that the large negative value of AuNP_RA_ indicates stable AuNPs [35]. Based on these results, the order of stability from highest to lowest stability of the AuNPs formed were AuNP_TET_ > AuNP_RA_ > AuNP_PAE_. The higher stability of AuNP_TET_ and AuNP_RA_ could be due to the purity of the samples, whereas AuNP_PAE_ was formed using the *P. aliciae* extract that contains a mixture of various compounds affecting the stability. The oxidation of polyhydroxy (-OH) groups contributes to nanoparticle stabilisation through the presence of negatively charged > C = O, which cap the surface of the Au through electrostatic interactions [36,37]. The -OH groups of tetracycline and rosmarinic acid could contribute to their higher stability. Nanoparticles formed at higher pH values are more likely to have higher ζ-potential values [38]. Both AuNP_TET_ and AuNP_RA_ were formed at high pH values of 11 and 9, respectively, which could explain their highly negative ζ-potential values. Although AuNP_PAE_ was less stable than AuNP_TET_ and AuNP_RA,_ it still displayed monodispersed nanoparticles and is considered stable according to the UV–vis analysis [39,40].

### 2.5. Dynamic Light Scattering (DLS) Analysis

**Figure 3:**
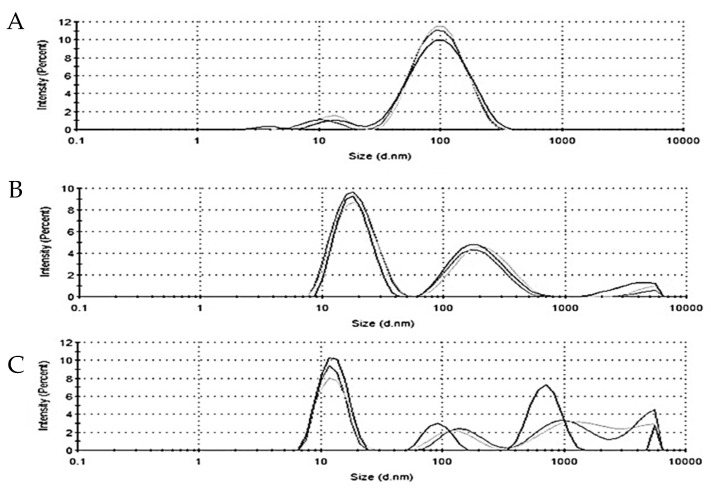
Hydrodynamic diameter of (**A**) *P. aliciae* gold nanoparticles (AuNP_PAE_), (**B**) rosmarinic acid gold nanoparticles (AuNP_RA_), and (**C**) tetracycline gold nanoparticles (AuNP_TET_), ***n*** = 3.

DLS is used to determine the hydrodynamic diameter of nanoparticles. The average hydrodynamic diameter of the AuNPs, AuNP_PAE_, AuNP_RA,_ and AuNP_TET_ was 71.26 ± 0.44 nm, 29.88 ± 3.30 nm, and 132.6 ± 99.5 nm, respectively (Figure 3). The larger diameter of AuNP_PAE_ could be due to the addition of gum arabic as a stabilising agent. 

### 2.6. Antibacterial Activity and Inhibition of Biofilm Development 

The antibacterial activity of the liquid AuNPs was investigated against *C. acnes* American Type Culture Collection 6919 (ATCC^®^ 6919), *S. epidermidis* (ATCC^®^ 35984), and a combination of these bacteria (*C. acnes + S. epidermidis*). *Cutibacterium acnes* and *S. epidermidis* have been associated with acne vulgaris, prosthetic-implant-associated infections, and wounds, either alone or in combination. These bacteria can form biofilms that contribute to antibiotic resistance since they act as a protective layer that prevents antibiotics from penetrating the biofilm and inhibiting the growth of the bacteria within [1,3,9].

No antibacterial activity was observed for AuNP_PAE_ and AuNP_RA_ at their highest concentrations tested of 25% *v*/*v* (≈777.5 µg/mL and ≈29.2 µg/mL µg/mL, respectively). This reduction in antibacterial activity could be due to the decrease in stability in BHI broth for these AuNPs, as seen in the stability studies (Figure S1). The current study showed no antibacterial activity of AuNP_TET_ at the highest concentration of 25% *v*/*v* (≈172.5 µg/mL) against *C. acnes* (ATCC^®^ 6919) and *C. acnes + S. epidermidis* grown aerobically. The antibacterial bacterial activity of AuNP_TET_ was observed at an MIC value of 0.39% *v*/*v* (≈0.67 µg/mL) against *S. epidermidis* (ATCC^®^ 35984) and *C. acnes* + *S. epidermidis* grown anaerobically (Table 1). The results are in accordance with the results obtained in an antibacterial study of AuNP_TET_. The researchers confirmed AuNP_TET_ had no antibacterial activity against tetracycline-resistant *Staphylococcus aureus* and *Escherichia coli*. Furthermore, the antibacterial activity of AuNP_TET_ displayed a higher MIC value than the tetracycline control against all the bacterial species tested [41]. Rosmarinic acid had no antibacterial activity at the highest concentration tested of 500 µg/mL against the tested bacteria. In comparison, PAE displayed antibacterial activity at MIC values of 7.8 µg/mL against *C. acnes* (ATCC^®^ 6919), 500 µg/mL against *S. epidermidis* (ATCC^®^ 35984), and 500 µg/mL against *C. acnes + S. epidermidis* grown under anaerobic and aerobic conditions (Table 1). Tetracycline displayed MIC values of 1.56 µg/mL against aerobically grown *C. acnes* + *S. epidermidis*. The MIC values observed for the tetracycline control were 0.78 µg/mL against *C. acnes*, *S. epidermidis*, and anaerobically grown *C. acnes* + *S. epidermidis*. Compounds with an MIC value lower than 16 µg/mL are considered to have noteworthy antibacterial activity [42]. 

No inhibition of biofilm development was observed for RA at the highest concentration tested of 500 µg/mL. PAE inhibited biofilm development at IC_50_ values of 3.2 ± 2.0 µg/mL (selectivity index; SI = 2.4), 231.9 ± 13.2 µg/mL (SI = 2.2), 25.7 ± 5.5 µg/mL (SI = 19.4) and 147.1 ± 7.6 µg/mL (SI = 3.4) against *C. acnes*, *S. epidermidis* and *C. acnes + S. epidermidis* grown anaerobically and aerobically, respectively (Table 1). Tetracycline prevented biofilm development at IC_50_ values of 0.9 ± 0.5 µg/mL, 1.6 ± 0.3 µg/mL, 0.9 ± 0.2 µg/mL, and 1.7 ± 0.7 µg/mL against *C. acnes*, *S. epidermidis*, and anaerobically and aerobically grown *C. acnes + S. epidermidis*, respectively (Table 1). However, the SI values for tetracycline were below one. A SI larger than one is an indication that the sample is targeting biofilm development [43]. Therefore, PAE can potentially prevent the biofilm development of acne-causing bacteria and, as a result, target antibiotic resistance associated with the disease when considering its SI values. No inhibition of biofilm development was observed for AuNP_PAE_ and AuNP_RA_ at their highest concentrations tested of 25% *v*/*v* (≈777.5 µg/mL and ≈29.2 µg/mL µg/mL, respectively). AuNP_TET_ prevented the development of *C. acnes* and *S. epidermidis* biofilms at IC_50_ values of 0.50 ± 0.049% *v*/*v* (≈0.86 ± 0.1 µg/mL) and 0.83 ± 0.013% *v*/*v* (≈1.43 ± 0.02), respectively. The inhibition of biofilm development of AuNP_TET_ was observed for anaerobically and aerobically grown *C. acnes + S. epidermidis* with IC_50_ values of 1.98 ± 2.25% *v*/*v* (≈3.42 ± 3.9 µg/mL) and 12.47 ± 1.32% *v*/*v* (≈21.51 ± 2.3 µg/mL), respectively. A SI larger than one was observed for AuNP_TET_ against *C. acnes* and aerobically grown *C. acnes + S. epidermidis*. However, biofilm development was not inhibited at a significantly lower concentration by AuNP_TET_ than the tetracycline control. The HAuCl_4_.3H_2_O control (HAuCl_4_) showed no antibacterial activity or inhibition of biofilm development. 

### 2.7. Cytotoxicity

The cytotoxicity of the respective AuNPs was tested on the human keratinocyte cell line, HaCaT. The cytotoxicity of the samples was tested to determine whether they could potentially be biocompatible with human keratinocytes considering that they would be topically applied. AuNP_PAE_, AuNP_RA_, and AuNP_TET_ displayed no cytotoxicity at the highest concentration tested of 25% *v*/*v* (≈777.5 µg/mL, ≈29.2 µg/mL and ≈172.5 µg/mL, respectively). The tetracycline compound and PAE alone displayed IC_50_ values of 185.4 ± 6.93 µg/mL and 65.16 ± 7.30 µg/mL, respectively. No cytotoxicity was observed for rosmarinic acid on HaCaT cells. Since no cytotoxicity was observed for the AuNPs, the samples were tested for their wound healing potential in vitro using HaCaT cells.

### 2.8. Wound Healing Potential

**Figure 4:**
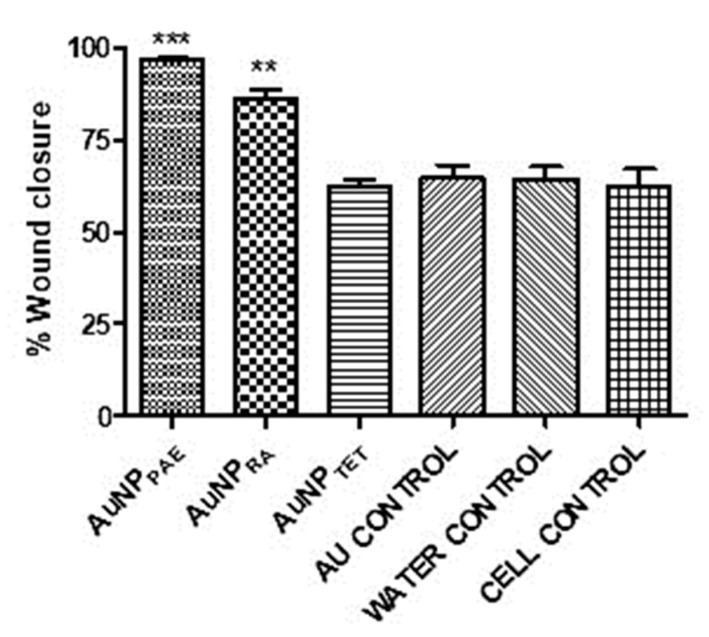
Percentage wound closure at 25% *v*/*v*. AuNP_PAE_: gold nanoparticles formed from *Plectranthus aliciae* ethanolic extract (≈777.5 µg/mL); AuNP_RA_: gold nanoparticles formed from rosmarinic acid (≈29.2 µg/mL; AuNP_TET_: gold nanoparticles formed from tetracycline (≈172.5 µg/mL); Au control: unreacted HAuCl_4_.3H_2_O control. One-way ANOVA followed by Tukey’s multiple comparison test, ***n*** = 3. ** *p* < 0.01, *** *p* < 0.001.

**Figure 5:**
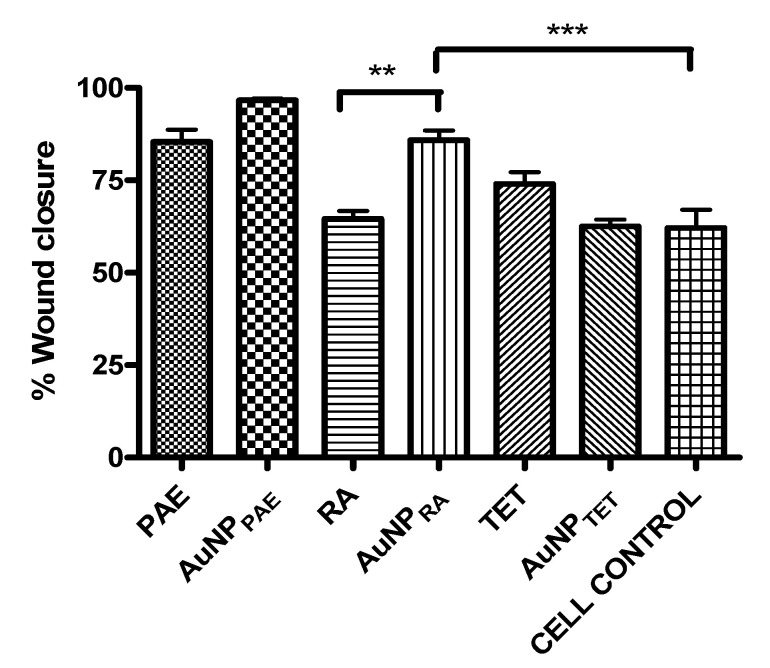
Percentage wound closure comparison between gold nanoparticle samples and their respective controls. AuNP_PAE_: gold nanoparticles formed from *Plectranthus aliciae* ethanolic extract (PAE); AuNP_RA_: gold nanoparticles formed from rosmarinic acid (RA); AuNP_TET_: gold nanoparticles formed from tetracycline (TET). One-way ANOVA followed by Tukey′s multiple comparison test, ***n*** = 3. ** *p* < 0.01, *** *p* < 0.001.

The respective AuNPs were tested in vitro as a preliminary study to determine their potential to promote wound closure. Sterile 25% *v*/*v* Aq. dest. was added to seeded HaCaT cells and served as the 100% closure control since all the nanoparticles were synthesised in Aq. dest. The percentage wound closure of the AuNPs compared to the Aq. dest. control is shown in Figure 4 and Table 2. AuNP_PAE_ displayed a significant percentage wound closure of 96.7 ± 1.0% (*p* < 0.001) at 25% *v*/*v* (≈777.5 µg/mL). PAE displayed a significant wound closure of 85.3 ± 7.4% at a 7.8 µg/mL concentration. Comparing the wound closure activity of PAE and AuNP_PAE,_ no significant improvement in wound closure was observed (Figure 5, Table 2). These results could indicate that at a lower concentration, the wound closure activity of AuNP_PAE_ could potentially decrease. No significant wound closure activity was observed for AuNP_TET_ (*p* > 0.05) (Table 2). 

AuNP_RA_ displayed significant wound closure activity (*p* < 0.01) with a percentage wound closure of 85.9 ± 5.9% at 25% *v*/*v* (≈29.2 µg/mL). No wound closure was observed for RA at the highest concentration tested of 500 µg/mL. Therefore, the wound healing activity of rosmarinic acid was significantly increased (*p* < 0.01) when the AuNP was capped with the compound (Figure 5, Table 2). Previous studies have identified the wound healing activity of polyphenolic compounds such as rosmarinic acid. Due to its high antioxidant and proinflammatory activity that results in the recruitment of inflammatory cells to the wound site, the polyphenolic compound rosmarinic acid has been proven to contribute to wound healing. The wound healing activity of polyphenols is due to these compounds contributing to the proliferation and migration of fibroblasts and endothelial cells to the wound site. However, polyphenolic compounds are unstable compounds under certain physiological conditions. Nanoparticles formed with polyphenolic compounds have shown increased activity compared to these compounds alone due to stabilising and controlled release of the compound at the wound site [14,44,45]. Therefore, polyphenolic compounds′ controlled release and ability to promote the migration of fibroblasts and endothelial cells to the wound site could explain the increased wound healing activity of AuNP_RA_ compared to RA. The wound closure activity of AuNP_RA_ contributes to the novelty of the current study.

## 3. Materials and Methods

### 3.1. Plectranthus aliciae Ethanolic Extraction 

*Plectranthus aliciae* plant material was collected from the Manie van der Schijff Botanical Garden at the University of Pretoria with the help of the garden curator Mr. Jason Sampson. Further taxonomical species identification was made with the help of Ms Magda Nel from the H. G. W. J. Schweickerdt Herbarium at the University of Pretoria, and a voucher specimen number was deposited (PRU 122336).

Leaves and soft twigs of *P. aliciae* were collected, making sure to remove hard wooden twigs. After collection, the plant material was rinsed with Aq. dest. and it was allowed to air dry for 1 h before it was dried at 40 °C in an oven for approximately 48 h or until dry. The dried material was ground with a 0.22 mm sieve using an IKA MF 10 basic grinder to a fine powder. The ground material was extracted using 99% ethanol (1:50 ratio of plant material to ethanol) for 72 h on a shaker. The extracted material was filtered using vacuum filtration and Whatman^®^ 1.0 filter paper. The extract was subjected to reduced pressure using a Buchi-R200 Rotavapor until dry. To ensure that the PAE was completely dry, it was dried to complete dryness at 40 °C for 24 h in an oven. The extract was stored at room temperature until further use. 

### 3.2. Synthesis of Gold Nanoparticles (AuNPs)

#### 3.2.1. Synthesis of *Plectranthus aliciae* Ethanolic Extract AuNPs (AuNP_PAE_)

In a 250 mL beaker, 100 mL of sterile Aq. dest. and 200 mg of PAE were heated to 90 °C for approximately 5 min. The solution was then transferred to 50 mL falcon tubes and centrifuged for 5 min at 980 rpm to remove any sedimentary particles. The supernatant was removed, and to 50 mL of the solution, 165 mg of gum arabic (GA) was added. The solution was then heated to 60 °C with constant stirring, and 8.25 mL of a 2.2 mM HAuCl_4_ solution was added and allowed to react for 30 min until nanoparticles were formed (solution became red wine colour). The solution was filtered through Whatman No. 1 filter paper, and the AuNP_PAE_ solution was stored at 2 °C until further use. The nano concentration was determined by drying 1 mL of AuNP_PAE_ at 40 °C until dry and recording the weight of the sample (***n*** = 3). 

#### 3.2.2. Synthesis of Rosmarinic Acid (RA) AuNPs (AuNP_RA_)

AuNP_RA_ were synthesised by dissolving 7.2 mg of RA in 100 mL sterile Aq. dest. at room temperature (RT). The pH of the solution was adjusted to 9 using a 1 M NaOH solution. To the solution, 15 mL of a 2.5 mM HAuCl_4_ solution was added and stirred for 30 min until nanoparticles formed, indicated by a red wine colour change. The solution was filtered through Whatman No. 1 filter paper, and the formed nanoparticle solution was stored at 2 °C until further use. The nano concentration was determined by drying 1 mL of AuNP_PAE_ at 40 °C until dry and recording the weight of the sample (***n*** = 3). 

#### 3.2.3. Synthesis of Tetracycline AuNPs (AuNP_TET_)

AuNP_TET_ was synthesised by dissolving 60 mg of TET in 60 mL of sterile Aq. dest. The pH of the solution was adjusted to 11 with 1 M NaOH. With constant stirring, 40 mL of a 1 mM HAuCl_4_ solution was added, and the solution was left to stir at RT for 24 h. After nanoparticle formation was observed, the solution was filtered through Whatman No. 1 filter paper, and the filtrate was stored at 2 °C until further use. The nano concentration was determined by drying 1 mL of AuNP_PAE_ at 40 °C until dry and recording the weight of the sample (***n*** = 3). 

### 3.3. Characterisation of Synthesised AuNPs

Characterisation studies were performed as described by De Canha et al. [46]. The formed AuNPs were analysed using various analytical instruments. The formation and stability of the AuNPs were analysed using an ultraviolet–visible (UV–vis) BIO-TEK Power-Wave XS multiwell plate reader (A.D.P., Weltevreden Park, RSA). Fourier transform infrared spectroscopy (FTIR; Perkin Elmer Spectrum 100) was used to determine the functional groups of compounds and extract bound to the AuNPs. The formation of stable AuNPs was determined using a Zetasizer Nano ZS (Malvern, United Kingdom) to measure the zeta (ζ) potential. The hydrodynamic diameter of the synthesised AuNPs was measured using dynamic light scattering spectroscopy (DLS). The morphological features and d-spacing of the synthesised AuNPs were determined using a JEOL JEM2100LaB6 HRTEM operated at 200 keV; SAED was also performed. 

### 3.4. Stability Studies of Synthesised AuNPs

The stability studies were conducted in brain heart infusion broth (BHI; Oxoid Thermo Fisher), Dulbecco’s Modified Eagle Medium (DMEM; supplemented with 1% gentamicin and 10% foetal bovine serum), 0.5% cysteine, 0.5% BSA, 5% sodium chloride (NaCl), and sterile Aq. dest. The nanoparticles were tested in a 1:2 ratio of AuNP to the tested solution. The ultraviolet absorbance of the nanoparticles was measured at 0 h, 2 h, 24 h (1 day), 48 h (2 days), 72 h (3 days), and 168 h (7 days) at a wavelength range of 450–800 nm using a Perkin Elmer VICTOR Nivo microplate reader. The samples were stored at 37 °C between analyses to mimic physiological conditions. 

### 3.5. Antibacterial Activity 

The antibacterial activity of the synthesised AuNPs were tested against *C. acnes* (ATCC^®^ 6919), *S. epidermidis* (ATCC^®^ 35984), and a combination of *C. acnes* (ATCC^®^ 6919) and *S. epidermidis* (ATCC^®^ 35984) (*C. acnes* + *S. epidermidis*) under anaerobic and aerobic growth conditions. The microdilution method was followed with slight modifications [47,48,49]. Briefly, bacterial cultures grown for 72 h on BHI agar were used to inoculate BHI broth with either *C. acnes*, *S. epidermidis,* or *C. acnes* + *S. epidermidis* to a final concentration of 1 × 10^6^ CFU/mL. In a 96-well plate, 100 µL of the synthesised AuNPs was serially diluted in brain heart infusion (BHI) broth with a concentration range of 0.20–25% *v*/*v*. Based on the weight determined during nanoparticle synthesis, the starting concentration of 25% *v*/*v* for each sample was ≈777.5 ± 4.3 µg/mL for AuNP_PAE_, ≈29.2 ± 3.8 µg/mL for AuNP_RA,_ and ≈172.5 ± 3.5 µg/mL for AuNP_TET_, respectively. The sample controls PAE, and the RA concentration ranged between 3.91 and 500 µg/mL. To each well of the test samples, 100 µL of the inoculated bacteria was added. The controls included tetracycline, which served as the positive control (0.39–50 µg/mL), Aq. dest., which served as the negative control (25% *v*/*v*), BHI broth, which served as the media control, a bacterial control of each tested bacterial species alone, and a 0.4 mM HAuCl_4_ solution control that served as a maximum gold effect to equate for potential unreactive gold in the AuNPs. The plates were incubated for 72 h at 37 °C under anaerobic conditions for *C. acnes*, aerobic conditions for *S. epidermidis*, and both anaerobic and aerobic conditions for *C. acnes* + *S. epidermidis*. After 72 h, 20 µL of PrestoBlue^®^ reagent (Thermo Fisher, Waltham, MA, USA) was added to all the wells and incubated for 60 min at 37 °C. The viability of the bacteria was determined visually through a colour change from blue to pink in the presence of metabolically active bacteria, and the minimum inhibitory concentration (MIC) was recorded. 

### 3.6. Inhibition of Biofilm Development

The inhibitory effect of the synthesised AuNPs on biofilm development was conducted with slight modifications [50]. The microdilution method was followed as described in Section 3.5. After 72 h of incubation, the supernatant was carefully removed, and the biofilms were washed once with Aq. dest. The plates were oven-dried, and the biofilm fixed with cold 99% methanol for 15 min. After removing the methanol, the plates were oven-dried again, and after the plates were dry, a 0.5% crystal violet (CV) solution was added to all the wells and incubated at room temperature for 20 min. The plates were then washed with Aq. dest. until the water was clear. The washed plates were dried, and the bound CV was dissolved using a 33% acetic acid solution for 20 min while shaking at 150 rpm. The destained biofilm solutions were transferred (100 µL) to clean 96-well plates. The optical density was measured at 590 nm using a Perkin Elmer VICTOR Nivo microplate reader. The 50% biofilm development inhibition concentration was determined using GraphPad Prism 4.0 software.

A SI was used to determine if the samples were more selective towards inhibiting the bacteria (antibacterial activity) or bacterial development. A SI larger than one is an indication that the sample is more selective as an inhibitor of bacterial development [43]. 

The SI was calculated using the following equation [43]:

$$\mathrm{Selectivity}\;\mathrm{Index}\;\left(\mathrm{SI}\right)=\;\frac{\mathrm{Minimum}\;\mathrm{inhibatory}\;\mathrm{concentration}\;\left(\mathrm{MIC}\right)}{\mathrm{Fifty}\;\mathrm{percent}\;\mathrm{biofilm}\;\mathrm{developmet}\;\mathrm{inhabtory}\;\mathrm{concentration}\;\left(\mathrm{BD}\right)}$$

### 3.7. Cytotoxicity

Human keratinocytes (HaCaT) were obtained from Dr. Lester Davids from the Department of Human Biology, the University of Cape Town [48]. The cells were grown in T75 culture flasks in DMEM supplemented with 100 µg/mL penicillin, 100 µg/mL streptomycin, 250 µg/mL fungizone, and 10% heat-inactivated FBS at 37 °C, 5% CO_2_ until a confluent monolayer formed. Trypsin-EDTA was used to detach the cells for no longer than 2 min and deactivated with fresh DMEM. The detached cells were seeded (1.0 × 10^5^ cells/mL; 100 μL) in the centre wells of a sterile 96-well plate and incubated for 24 h at 37 °C, 5% CO_2_. Stock solutions of the samples were prepared in 24-well plates in DMEM, and 100 µL of the sample dilutions were transferred to the 96-well plates containing the cells. The final concentrations of the liquid AuNP samples ranged between 0.20 and 25% *v*/*v* and between 3.12 and 400 µg/mL for PAE, RA, and TET. Actinomycin D was included in the study as a positive control with a final concentration range of 0.05–3.91 × 10^−4^ µg/mL. A 0.4 mM HAuCl_4_ solution was included in the study as a maximum gold effect to equate for potential unreactive gold in the AuNPs. A 25% *v*/*v* sterile Aq. dest. to DMEM with cells control and media control without cells were also included in the experiment and served as the negative control. The plates were incubated for 72 h at 37 °C with 5% CO_2_. After incubation, 20 µL of PrestoBlue^®^ reagent was added, and the plates were incubated for 2 h. The fluorescence was measured at excitation (560 nm) and emission (590 nm) wavelengths using a Perkin Elmer VICTOR Nivo microplate reader. 

### 3.8. Wound Healing Potential

The scratch assay was conducted with slight modifications [51]. To the wells of a 48-well plate, 500 µL of HaCaT cells was seeded to a concentration of 7.5 × 10^4^ cells per well in DMEM supplemented with 100 µg/mL penicillin, 100 µg/mL streptomycin, 250 µg/mL fungizone, and 10% FBS. The plates were incubated for 24 h at 37 °C with 5% CO_2_ until a confluent monolayer formed. A cross was made from top to bottom and left to right using a sterile 1000 µL tip in each well. The media was carefully removed, the wells were washed once with sterile phosphate buffered saline (PBS), and 1125 µL of fresh DMEM was added. Pictures of the untreated scratched wells containing the cells were taken under a ZEISS Primovert microscope at a four-time magnification (phase 0) that served as the pre-treatment control (0 h). The samples were added to the wells of the 48-well plate to a final volume of 1500 µL and a final sample starting concentration of 25% *v*/*v* for the AuNPs and 0.5MIC for PAE, RA, and TET (Table 1). A 0.4 mM HAuCl_4_ solution was included in the study as a maximum gold effect to equate for potential unreactive gold in the AuNPs. A 25% *v*/*v* sterile Aq. dest. control with cells and media control without cells were included in the experiment. The plates were then incubated at 37 °C, 5% CO_2_ for 18 h. After incubation, a picture was taken of each well (Nikon camera D90). The percentage wound closure was calculated using ImageJ and GraphPad Prism 4 software. The viability of the cells for each sample was determined by first removing 1300 µL of the sample from the wells and adding 20 µL of PrestoBlue^®^ reagent to each of the wells. The fluorescence was measured using a Perkin Elmer VICTOR Nivo microplate reader (excitation: 560 nm and emission: 590 nm wavelengths).

The following formula was used to determine the percentage wound closure from 0 h to 18 h where “A” is the area of the scratch [52]:

$$\%\;\mathrm{Wound}\;\mathrm{closure}=\left(\frac{A0h-A18h}{A0h}\right)\;\times 100\%$$

### 3.9. Statistical Analysis

All in vitro biological experiments were performed in triplicate (***n*** = 3) and in three independent experiments to ensure reproducible results. The 50% inhibitory concentration (IC_50_) and statistical analyses were conducted using GraphPad Prism 4 software. The statistical significance of the results was determined with a one-way analysis of variance (ANOVA) and Tukey′s multiple comparison test with * *p* < 0.05, ** *p* < 0.01, and *** *p* < 0.001 results regarded as statistically significant.

## 4. Conclusions

The study aimed to investigate the antibacterial and wound healing potential of biosynthesised AuNP of *P. aliciae,* rosmarinic acid, and tetracycline as potential treatments for acne vulgaris, prosthetic-implant-associated infections and wounds. 

Gold nanoparticles could hold the key to improved activity of plant extracts and their compounds. Gold nanoparticles from *P. aliciae*, rosmarinic acid, and tetracycline were successfully formed. Although the biosynthesised AuNPs had no improved antibacterial or inhibition of biofilm development, significant wound healing activity was observed for AuNP_RA._ The AuNP_RA_ is potentially a safe and effective nano treatment for wounds due to its low cytotoxicity and significantly increased wound healing activity. Further studies could investigate the in vivo wound healing activity and the permeability of AuNP_RA_. This is the first-time report of the wound healing potential of AuNP_RA,_ which contributes to the novelty of the study. Based on the results obtained in this study, in vivo wound healing studies will be the next step toward developing a wound healing technology of rosmarinic acid formed gold nanoparticles.

## Figures and Tables

**Table 1:** Antibacterial and biofilm development inhibitory concentration of *Plectranthus aliciae* ethanolic extract (PAE), *P. aliciae* gold nanoparticles (AuNP_PAE_), rosmarinic acid (RA), rosmarinic acid gold nanoparticles (AuNP_RA_), tetracycline, and tetracycline gold nanoparticles (AuNP_TET_). The antibacterial activity was determined against *Cutibacterium acnes* (ATCC^®^ 6919), *Staphylococcus epidermidis* (ATCC^®^ 35984), and a combination of these bacteria under anaerobic and aerobic growth conditions.

SINGLE SPECIES SYSTEM					MULTISPECIES SYSTEM				
Strain	Sample	MIC ^a^ (µg/mL)	Inhibition Biofilm Development (BD) (IC_50_^b^ ± SD ^c^)	SI ^d^	Strain	Sample	MIC ^a^ (µg/mL)	Inhibition Biofilm Development (BD) (IC_50_^b^ ± D ^c^)	SI ^d^
*C. acnes* (ATCC^®^ 61919)	PAE	7.8	3.2 ± 2.0	2.4	*C. acnes* (ATCC^®^ 6919) + *S. epidermidis* (ATCC^®^ 35984) anaerobic growth	PAE	500	25.7 ± 5.5	19.4
	AuNP_PAE_	NI ^e^	NI ^e^	-		AuNP_PAE_	NI ^e^	NI ^e^	-
	RA	NI ^f^	NI ^f^	-		RA	NI ^f^	NI ^f^	-
	AuNP_RA_	NI ^e^	NI ^e^	-		AuNP_RA_	NI ^e^	NI ^e^	-
	Tet	0.78	0.9 ± 0.5	0.9		Tet	0.78	0.9 ± 0.2	0.9
	AuNP_TET_	NI ^f^	0.86 ± 0.1	200		AuNP_TET_	0.67	3.42 ± 3.9	0.2
*S. epidermidis* (ATCC^®^ 35984)	PAE	500	231.9 ± 13.2	2.2	*C. acnes* (ATCC^®^ 6919) + *S. epidermidis* (ATCC^®^ 35984) aerobic growth	PAE	500	147.1 ± 7.6	3.4
	AuNP_PAE_	NI ^e^	NI ^e^	-		AuNP_PAE_	NI ^e^	NI ^e^	-
	RA	NI ^f^	NI ^f^	-		RA	NI ^f^	NI ^f^	-
	AuNP_RA_	NI ^e^	NI ^e^	-		AuNP_RA_	NI ^e^	NI ^e^	-
	Tet	0.78	1.6 ± 0.3	0.5		Tet	1.56	1.7 ± 0.7	0.9
	AuNP_TET_	0.67	1.43 ± 0.02	0.5		AuNP_TET_	NI ^e^	21.51 ± 2.3	8.0

^a^ MIC: minimum inhibitory concentration; ^b^ IC_50_: 50% inhibitory concentration; ^c^ SD: standard deviation; ^d^ SI: selectivity index (SI = MIC/BD); ^e^ NI: no inhibition at the highest concentration tested of 25% *v*/*v*; ^f^ NI: no inhibition at the highest concentration tested of 500 µg/mL.

**Table 2:** Cytotoxicity and percentage wound closure of *Plectranthus aliciae* ethanolic extract (PAE), *P. aliciae* gold nanoparticles (AuNP_PAE_), rosmarinic acid (RA), rosmarinic acid gold nanoparticles (AuNP_RA_), tetracycline, and tetracycline gold nanoparticles (AuNP_TET_) compared to the cell growth, distilled water, and gold salt controls.

Sample	Cytotoxicity on HaCaT Cells (IC_50_^a^ ± SD ^b^)	% Wound Closure (%Closure ± SD ^a^)
PAE	65.16 ± 7.30	85.3 ± 7.4
AuNP_PAE_	NI ^c^	96.7 ± 1.0
RA	NI ^d^	64.6 ± 4.4
AuNP_RA_	NI ^c^	85.9 ± 5.9
Tet	185.4 ± 6.93	74.1 ± 6.3
AuNP_TET_	NI ^c^	62.6 ± 4.0
Cell control	-	62.1 ± 11.0
Distilled water control	-	64.5 ± 5.5
Gold control	-	64.6 ± 7.2

^a^ IC_50_: 50% inhibitory concentration; ^b^ SD: standard deviation; ^c^ NI: no inhibition at the highest concentration tested of 25% *v*/*v*; ^d^ NI: no inhibition at the highest concentration tested of 500 µg/mL.

## Data Availability

Data is contained within the article and supplementary material.

## Supplementary Materials

The following supporting information can be downloaded at: https://www.mdpi.com/article/10.3390/ph15080933/s1, Figure S1: Ultraviolet-visible spectroscopy (UV-Vis) and stability studies of biosynthesised gold nanoparticles; Table S1: Functional groups identified with Fourier Transform Infrared Spectroscopy.
