# Supplementary material for: Targeting Acne Bacteria and Wound Healing In Vitro Using Plectranthus aliciae, Rosmarinic Acid, and Tetracycline Gold Nanoparticles
Source: Pharmaceuticals (Basel). 2022 Jul 28;15(8):933. doi: 10.3390/ph15080933 (PMC9412649; doi:10.3390/ph15080933)
Supplement: Supplementary file 1 [file pharmaceuticals-15-00933-s001.zip › pharmaceuticals-1769614-supplementary.pdf]

*pharmaceuticals*

MDPI

*Article: Supplementary Material*

# Targeting Acne Bacteria and Wound Healing In Vitro Using *Plectranthus aliciae*, Rosmarinic Acid, and Tetracycline Gold Nanoparticles

**Isa A. Lambrechts [1], Velaphi C. Thipe [2], Kattesh V. Katti [2], Vusani Mandiwana [3], Michel Lonji Kalombo [3], Suprakas Sinha Ray [4], Rirhandzu Rikhotso [4], Arno Janse van Vuuren [5], Tenille Esmear [1] and Namrita Lall [1,6,7,*]**

1 Department of Plant and Soil Sciences, Faculty of Natural and Agricultural Sciences, University of Pretoria, Hatfield, Pretoria 0002, South Africa; u29021759@tuks.ac.za (I.A.L.); tenille.esmear@gmail.com (T.E.)

2 Department of Radiology, Institute of Green Nanotechnology, University of Missouri Columbia, Columbia, MO 65212, USA; vctqnf@health.missouri.edu (V.C.T.); kattik@health.missouri.edu (K.V.K.)

3 Chemical Cluster, Centre for Nanostructures and Advanced Materials, Council for Scientific and Industrial Research, Pretoria 0184, South Africa; vusy.mandi@gmail.com (V.M.); lkalombo@csir.co.za (M.L.K.)

4 DST/CSIR National Centre for Nanostructured Materials, Council for Scientific and Industrial Research, Pretoria 0184, South Africa; rsuprakas@csir.co.za (S.S.R.); rrikhotso@csir.co.za (R.R.)

5 Centre for High-Resolution Transmission Electron Microscopy, Nelson Mandela University, Port Elizabeth 6001, South Africa; arno.jansevanvuuren@mandela.ac.za

6 School of Natural Resources, College of Agriculture, Food and Natural Resources, University of Missouri, Columbia, MO 65211, USA

7 College of Pharmacy, JSS Academy of Higher Education and Research, Mysuru 570015, India

* Correspondence: namrita.lall@up.ac.za

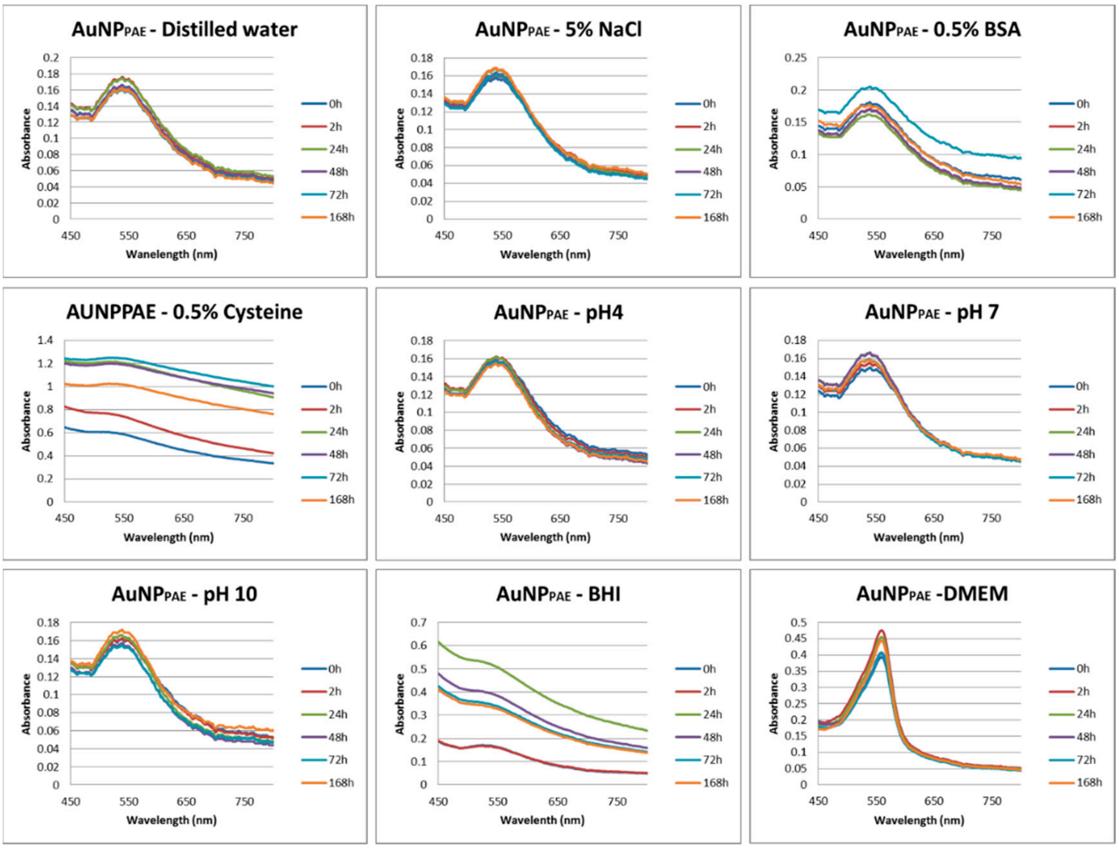


**(A)**

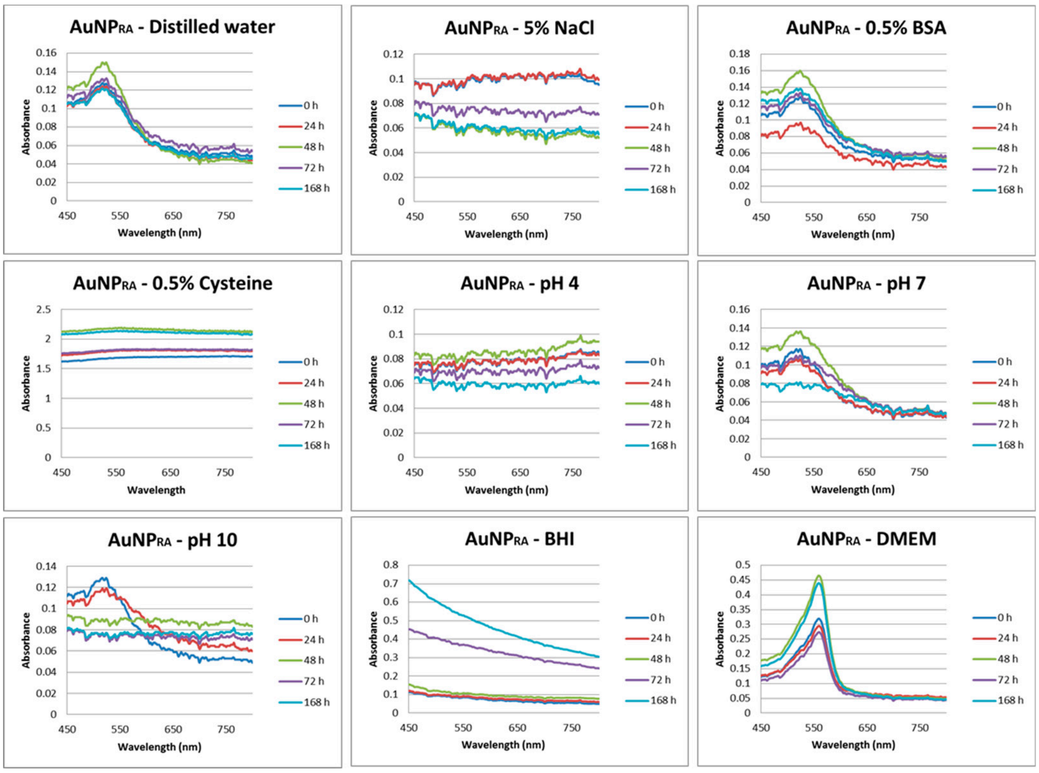


(**B**)

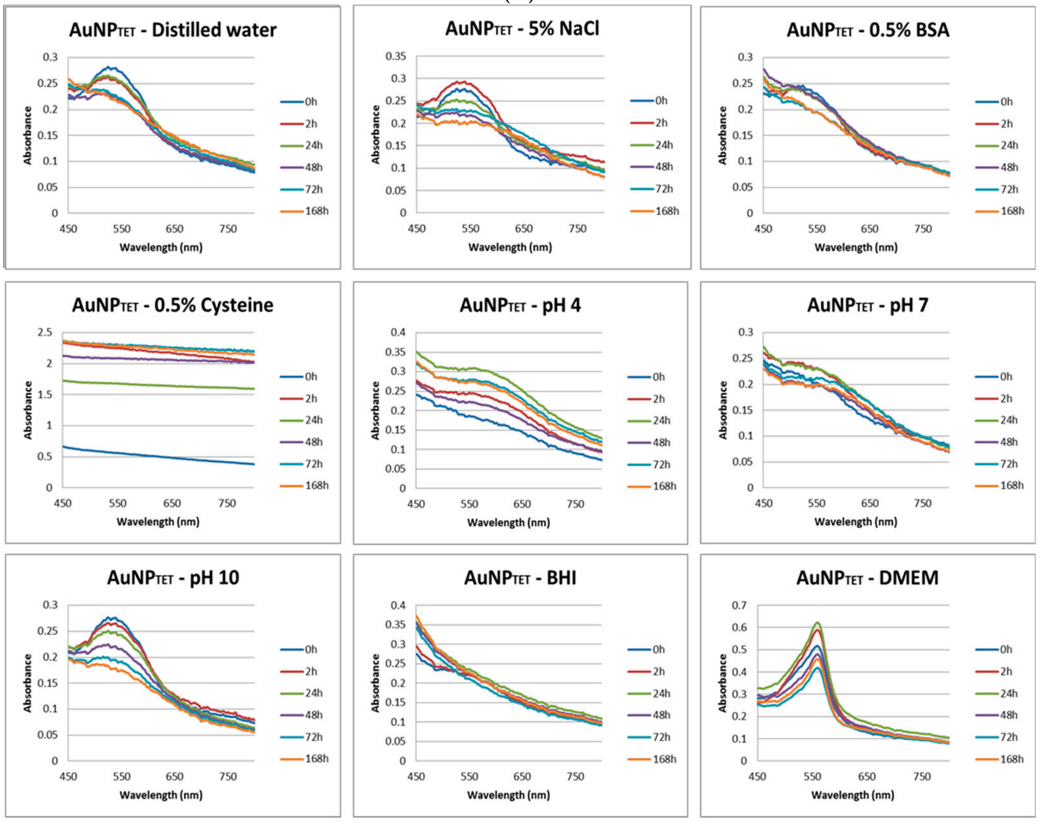


(**C**)

Figure S1: Ultraviolet-visible spectroscopy (UV-Vis) and stability studies of biosynthesised gold nanoparticles (AuNPs) at various time intervals under various physiological conditions. (**A**) Biosynthesized Plectranthus aliciae AuNPs (AuNPPAE); (**B**) biosynthesised rosmarinic acid AuNPs (AuNPRA); (**C**) biosynthesised tetracycline AuNPs (AuNPTET)in NaCl: sodium chloride; BSA: bovine serum albumin; BHI: brain heart infusion broth; DMEM: Dulbecco's Modified Eagle Medium.

Table S1: Functional groups identified with Fourier Transform Infrared Spectroscopy.

| **Sample** | **Control** | | **AuNP** | | **Group (stretching)** | **Compound class** | **Reference** |
|---|---|---|---|---|---|---|---|
| | **WL ($cm^{-1}$)** | **% T** | **WL ($cm^{-1}$)** | **% T** | | | |
| ***Plectranthus aliciae*** | 3728 | 98.30 | 3727 | 98.15 | O-H stretching | Free alcohol | [33,34] |
| | 3361 | 80.49 | 3272 | 48.85 | N-H stretching | Aliphatic primary amine | [33,34] |
| | 2360 | 80.73 | 2360 | 87.08 | O=C=O stretching | Carbon dioxide | [34,35] |
| | 2342 | 85.04 | 2342 | 89.61 | O=C=O stretching | Carbon dioxide | [34,35] |
| | 2075 | 98.49 | 2075 | 95.69 | N=C=S stretching | isothiocyanate | [33,34] |
| | 1635 | 83.37 | 1636 | 70.84 | C=C OR N-H bending | Alkene OR amine | [33,34] |
| **Rosmarinic acid** | 3772 | 95.82 | 3772 | 98.64 | O-H stretching | Free alcohol | [33,34] |
| | 3302 | 77.01 | 3302 | 48.77 | O-H stretching | Carboxylic acid | [33,34] |
| | 2360 | 70.61 | 2360 | 76.02 | O=C=O stretching | Carbon dioxide | [34,35] |
| | 2342 | 76.24 | 2342 | 80.97 | O=C=O stretching | Carbon dioxide | [34,35] |
| | 1647 | 71.90 | 1636 | 70.85 | C=C stretching | alkene | [34] |
| | 1348 | 46.25 | 1356 | 91.10 | O-H bending | phenol | [34] |
| | 1259 | 34.69 | 1253 | 90.95 | C-O | aromatic ester | [34] |

| | | | | | | | |
|---|---|---|---|---|---|---|---|
| | 1152 | 23.96 | 1150 | 90.92 | C-O stretching | aliphatic ether | [33,34] |
| | 669 | 52.34 | 668 | 39.41 | C=C bending | alkene | [34] |
| **Tetracycline** | 3728 | 91.06 | 3728 | 95.89 | O-H stretching | free alcohol | [33] |
| | 3705 | 91.90 | 3696 | 95.64 | O-H stretching | Free alcohol | [33] |
| | 3626 | 90.43 | 3623 | 85.22 | O-H stretching | Free alcohol | [33] |
| | 3301 | 82.30 | 3304 | 48.62 | N-H stretching | aliphatic primary amine | [33,34] |
| | 2361 | 57.06 | 2361 | 64.48 | O=C=O stretching | Carbon dioxide | [34,35] |
| | 2342 | 64.48 | 2342 | 71.77 | O=C=O stretching | Carbon dioxide | [34,35] |
| | 1641 | 75.89 | 1635 | 70.77 | C=C or N-H bending | cyclic alkene or amine | [34] |
| | 1409 | 90.79 | 1393 | 65.60 | O-H bending | alcohol | [33,34] |
| | 1252 | 90.72 | 1255 | 65.35 | C-O stretching | aromatic ester or alkyl aryl ether | [33,34] |
| | 1161 | 90.63 | 1176 | 65.45 | C-N stretching | amine | [33,34] |
| | 1060 | 90.32 | 1065 | 71.41 | C-O stretching | primary alcohol | [33,34] |
| | 669 | 53.99 | 669 | 37.81 | C=C bending | alkene | [34] |
